# An Important Dento-Legal Function

**Published:** 1898-12

**Authors:** A. L. Benedict

**Affiliations:** Professor of Physiology and Digestive Diseases, Dental Department, University of Buffalo.


ARTICLE IX.

### AN IMPORTANT DENTO-LEGAL FUNCTION.

A. L. BENEDICT, A. M., M. D.

Professor of Physiology and Digestive Diseases, Dental Department, University of Buffalo.

It has lately happened that the mutilated fragments of a human body have been several times identified, each time with great positiveness, as belonging to as many different individuals, One means of identification, which seemed both scientific and absolute, was a record kept by a dentist, the peculiarities of a filling being described in detail. Notwithstanding this testimony, it was disproved by the appearance of the person to whom the body was said to have belonged.

Without wishing to criticise too severely the error of being sure when the facts are against one, it is wise for every professional man to form habits of exact observation, and, notwithstanding them, to avoid committing himself absolutely to an opinion. Not to dwell upon this lesson of the case cited, we must also deduce the importance of the dentist's testimony, when carefully given, in regard to legal questions as to human identity. Identification becomes legally important under several diverse circumstances, as follows: (1) The discovery of an unknown corpse when it is obvious that a crime has been committed; (2) Similarily, in the absence of indications of crime, when the disposition of the body is considered; (3) When self-identification is impossible on account of unconsciousness or lack of memory. Not only may acute disease, particularly with a high fever, and injuries of the head produce unconsciousness, but various mental diseases, including phases of insanity, must be considered. Not infrequently a business man of middle life. after a prolonged state of fatigue and worry, becomes temporarily insane and is actuated by the impulse to escape from his troubles. He leaves his home, and after days, weeks or months, is found, or, so to speak, finds himself, in a strange place, and with no recollection of the intervening time, or, at most, a very vague remembrance of some salient points of his mental aberration. The disappearance of a business man almost inevitably arouses suspicions of forgery, embezzlement or other dishonestly, or of an elopement, with the usual domestic complications. Only the most conspicuous absence of all such irregularities will shield the unfortunate individual and his almost equally unfortunate family from the stigma of social disgrace. The importance of identification under such circumstances can scarcely be overestimated. (4) Under a separate number may be mentioned impossibility of self-indentification from non-pathologic reasons, such as the inability of little children to communicate their personality, or similar inability from permanent disability, deaf-mutism or high grades of speech-imperfection, combined with inability to write. It would, perhaps, be too far-fetched to include the cases of foreigners unable to speak the language of the country, since it would be difficult to imagine a case in which it would not be easier to secure an interpreter than a dental expert familiar with the individual; (5) When, to avoid punishment, identity is denied; (6) When, as in the famous Tichborne case, a false identity is claimed for some purpose.

In any question of legal identification, the dental profession is placed in a position of peculiar influence and power. The ordinary recognition of acquaintances, even among relatives of the greatest intimacy, is by means of memory-pictures which can not be communicated to a third person, unless by an artist. Ordinary descriptions of hair, eyes, stature, deformities and blemishes are so vague as to be of almost no value for identification. In the case cited at the beginning of this article, a man supposed himself to be the father of the victim and identified the remains by a scar, yet the individual thus asserted to be dead proved an alibi by appearing in the flesh. The Bertillon system, however perfect, is not available except in the case of criminals already recorded. Ordinary physical measurements are not of much value. Photographs are very likely to be misleading and, in many instances, pains are taken to disguise or to mutilate the face. If the "Puddenhead Wilson" system of impressions of the thumb came into vague, accidental or intentional mutilation of the thumb would likewise interfere with its operation.

It might be thought that the physician would be able to supply needed marks of identification and, occasionally, his recollections are of assistance. Thus Formad, of Philadelphia, once identified the headless and limbless trunk of a negro by demonstrating a recent pleurisy, and then searching dispensary records till he found the name and address of the victim. Usually, however, diseases are too evanescent, or are not capable of exact localization. Moreover, the great majority of individuals seldom or never visit the physician, while most persons who are of recognizable value in society are rather regular in their visits to the dentist. While the physician's ministrations are for the purpose of removing blemishes which might lead to identification, the dentist is a surgeon, and his work always leaves a definite trace. A filling, a set of false teeth, a plate, a pivot tooth, an orthodontic apparatus, a drainage of the antrum, the removal of a tooth, all these demands on the skill of the dentist afford an admirable opportunity for registration of his observations, while the ingenuity of his profession has provided him with record books, so that the basis of a scientific identification is afforded by his business memoranda.

It is not the purpose of this article to emphasize the many financial and professional advantages to be derived from making accurate and complete notes of every case, but to point out that the highest possibilities of that part of medico-legal lore which deals with identification, lies in the development of one phase of dental science and art.—*Dental Review.*

---

